# Übersicht über die in der EU zugelassenen COVID-19-Impfstoffe – von der Technologie über die klinische Prüfung zur Zulassung

**DOI:** 10.1007/s00103-022-03600-4

**Published:** 2022-10-18

**Authors:** Eberhard Hildt

**Affiliations:** Abteilung Virologie, Paul-Ehrlich-Institut, Bundesinstitut für Impfstoffe und biomedizinische Arzneimittel, Paul-Ehrlich-Straße 51–59, 63225 Langen, Deutschland

**Keywords:** SARS-CoV-2, Plattformtechnologie, Immunantwort, Adjuvans, Nanopartikel, SARS-CoV2, Platform technology, Immune response, Adjuvant, Nanoparticle

## Abstract

Derzeit (Stand Juli 2022) sind in der EU 6 verschiedene COVID-19-Impfstoffe zugelassen. Diese umfassen 2 mRNA-basierte Impfstoffe (BNT162b2, Comirnaty® und mRNA-1273, Spikevax®), 2 auf einem adenoviralen Vektor basierende Impfstoffe (AZD1222, Vaxzevria® und Ad26.COV2.S, Jcovden®) sowie den Untereinheitenimpfstoff Nuvaxovid® (NVX-CoV2373) und den Inaktivatvirus-Impfstoff VLA2001. Obgleich diese Impfstoffe auf unterschiedlichen Technologien basieren, ist allen die Verwendung des Spike-Proteins von SARS-CoV‑2 als Antigen gemein.

Diese Übersicht beschreibt die Charakteristika ihrer Zusammensetzung, ihre Wirksamkeit und den Einfluss verschiedener Faktoren auf die Wirksamkeit. Des Weiteren wird das Zulassungsverfahren erläutert und die Faktoren werden identifiziert, welche zu der bisher noch nicht dagewesenen Schnelligkeit in der Entwicklung und Zulassung von Impfstoffen gegen einen pandemischen Erreger beigetragen haben.

## Abstract (en)

Currently (as of July 2022), six different COVID-19 vaccines are licensed in the EU. These include two mRNA-based vaccines (BNT162b2, Comirnaty® and mRNA-1273, Spikevax®), two adenoviral vector-based vaccines (AZD1222, Vaxzevria® and Ad26.COV2.S, Jcovden®), the subunit vaccine Nuvaxovid® (NVX-CoV2373), and the inactivated virus vaccine VLA2001. Although these vaccines are based on different technologies, they all share the use of the spike protein of SARS-CoV‑2 as antigen.

This overview describes the characteristics of their composition, their efficacy, and the impact of various factors on efficacy. Another aspect of this overview is the description of the approval process and the identification of factors that have contributed to the unprecedented speed in the development and approval of vaccines against a pandemic pathogen.

## Einleitung

Wesentliche Grundlage aller derzeit in der EU zugelassenen Impfstoffe gegen SARS-CoV‑2 ist das 1273 Aminosäuren (aa) umfassende Spike-Protein, das als Trimer vorliegt. Im Rahmen der natürlichen Infektion wird das Spike-Protein von einer subgenomischen viralen RNA ausgehend gebildet [1]. Aufgrund einer N‑terminalen Signalsequenz (aa1-13) erfolgt die Translokation in das Lumen des endoplasmatischen Retikulums (ER), wo die Abspaltung dieser Signalsequenz erfolgt. Die C‑terminale Transmembranregion verankert das Spike-Protein in der ER-Membran. Im Zuge der Freisetzung des Viruspartikels erfolgen N- und O‑Glykosylierungen und in der trans-Golgi-Region durch die Protease Furin eine proteolytische Spaltung in die membranassoziierte S2-Untereinheit und die äußere S1-Untereinheit. Die beiden Untereinheiten sind über nichtkovalente Wechselwirkungen miteinander verbunden.

Das Spike-Protein umfasst verschiedene Domänen: Neben der N‑terminalen Domäne (NTD) liegt auch die rezeptorbindende Domäne (RBD) in der S1-Region; in der S2-Untereinheit befindet sich die fusogene Sequenz. Auf der Oberfläche des Virus befinden sich ca. 15 bis 30 Homotrimere des Spike-Proteins [2].

Die Struktur des Spike-Proteins ist in einigen Bereichen sehr flexibel. So kann die RBD in unterschiedlichen Konformationen vorliegen. In der sog. Up-position kann die Interaktion mit dem Rezeptor ACE2 („angiotensin converting enzyme 2“) erfolgen. Das reife Spike-Protein liegt dabei in einer metastabilen Konformation vor. Für die Induktion einer neutralisierenden Antikörperantwort ist es erstrebenswert, dass das Protein in seiner nativen Konformation vorliegt und die RBD zugänglich ist. Um dies zu erreichen, erfolgt bei vielen Impfstoffkonzepten die Einführung der Mutationen K986P und V987P, die das Spike-Protein in einer Präfusionskonformation stabilisieren [3, 4].

Im Nachfolgenden soll nun auf die derzeit in der EU zugelassenen Impfstoffe eingegangen werden (Stand Juli 2022, Übersicht in Tab. 1), auf deren Zulassung und den Einfluss verschiedener Faktoren auf ihre Wirksamkeit. Der Aspekt der Sicherheit und Verträglichkeit dieser Impfstoffe wird in einem gesonderten Beitrag in diesem Themenheft untersucht (siehe Beitrag von Keller-Stanislawski).

**Table 1:** 

Name	Typ	Hersteller	Stabilisierende Mutationen	Datum der Zulassung	Dosis (Grundimmunisierung, Erwachsene)
BNT162b2 Comirnaty®	mRNA	BioNTech/Pfizer	Ja (pro Doppelmutation)	21.12.2020	2 × 30 µg
mRNA-1273 Spikevax	mRNA	Moderna	Ja (pro Doppelmutation)	06.01.2021	2 × 100 µg
AZD1222 Vaxzevria®	Adenoviral	Univ. Oxford/AstraZeneca	Nein	29.01.2021	2 × 5 × 10e10 adenovirale Partikel
Ad26.COV2.S Jcovden®	Adenoviral	Janssen/Cilag	Ja (pro Doppelmutation) und Furinspaltstelle	11.03.2021	1 × 5 × 10e10 adenovirale Partikel
NVX-CoV2373 Nuvaxovid®	Protein	Novavax	Ja (pro Doppelmutation) und Furinspaltstelle	20.12.2021	2 × 5 µg und Adjuvans
VLA2001	Inaktiviertes Virus	Valneva	Nein	24.06.2022	2 × 33 Antigeneinheiten und Adjuvans

## Impfstoffklassen

### mRNA-basierte Impfstoffe

Derzeit sind in der EU 2 mRNA-Impfstoffe gegen COVID-19 zugelassen, mRNA-1273 (Spikevax®) von Moderna und BNT162b2 (Comirnaty®) von BioNTech/Pfizer.

mRNA-basierte Impfstoffe setzen sich aus 2 Hauptkomponenten zusammen: die mRNA („drug substance“) und ein lipidbasiertes Verpackungssystem, das zugleich eine Adjuvansfunktion besitzt [5, 6]. Beide Impfstoffe enthalten eine Codon-optimierte RNA, die für das komplette Spike-Protein codiert. Beiden sind auch die Präfusionskonformation-stabilisierenden Mutationen K986P und V987P gemein. Die Herstellung der mRNA erfolgt ausgehend von einer DNA-Matrize (linearisiertes, Spike-Protein-codierendes Plasmid) durch In-vitro-Transkription. Um die Stabilität und Translatierbarkeit der mRNA zu erhöhen, werden optimierte 5′- und 3′-UTRs („untranslated regions“) sowie ein Poly-A-Schwanz am 3′-Ende und ein 5′-Cap verwendet. Der Ersatz von Uridin durch N1-Methylpseudouridine dient der Erhöhung der Stabilität, aber insbesondere auch dem Ziel, die durch die angeborene Immunantwort ausgelöste Reaktogenität zu vermindern. Dafür ist es ebenfalls erforderlich, den Anteil an doppelsträngiger RNA (dsRNA) durch Reinigung der mRNA weitestgehend zu entfernen [7–11].

Die Verpackung der RNA in Form sogenannter Lipidnanopartikel (LNP) dient 2 Zielen: Erstens sind RNAs instabile Moleküle, die leicht durch verschiedene Hydrolasen abgebaut werden können, sodass die „Verpackung“ eine Schutzfunktion ausübt. Zweitens steht die starke negative Gesamtladung der RNA einem effizienten Transfer über zelluläre Membranen in das Zellinnere entgegen. Die Verpackung in Form von LNP wirkt mithin analog einem Transfektionsreagenz und ermöglicht so den effizienten Transfer in Zellen (Muskelzellen, Fibroblasten, Immunzellen wie dendritische Zellen (DCs)), sodass es zur Proteinproduktion an der Injektionsstelle, in den drainierenden Lymphknoten und auch in der Milz kommt. Neben der Proteinproduktion in den DCs am Injektionsort und in den drainierenden Lymphknoten werden in diesen Zellen Typ-I-Interferone und eine Reihe von Zytokinen und Chemokinen gebildet.

Die genaue Zusammensetzung der LNP ist von entscheidender Bedeutung für die Effizienz der Verpackung und den Transfer der mRNA. Die Entwicklung geeigneter Lipidkomponenten und die Optimierung der Formulierung waren essenziell für die breite Anwendung von mRNA-basierten Impfstoffen. LNP bestehen aus einer Kombination kationischer Lipide, Phospholipiden, Cholesterol und Polyethylenglykol-(PEG-)konjugierter Lipide [12, 13]. Die kationischen Lipide komplexieren dabei die polyanionische RNA. Das Cholesterol beeinflusst die Fluidität der artifiziellen Membran und der PEG-Anteil hat einen Einfluss auf die Stabilität der LNP.

mRNA-basierte Impfstoffe sind nicht adjuvantiert. Beide Komponenten, sowohl die mRNA als auch die Lipidkomponenten, üben jedoch eine immanente Adjuvansfunktion aus. mRNAs können durch Pattern-Recognition-Rezeptoren (PRR) in der Zelle erkannt werden. So erkennen Toll-like-Rezeptoren (TLR)3, TLR7 und TLR9 sowohl dsRNA als auch einzelsträngige (ss)RNA, während TLR8 ausschließlich ssRNA erkennt. Weitere RNA-Sensoren sind RIG‑I, MDA‑5 und LGP2. Die Lipidkomponenten wirken als Adjuvans, indem sie die Produktion inflammatorischer Zytokine und Chemokine wie Interleukin (IL-) 1β, IL‑6, CC-Chemokinligand (CCL)3 und CCL4 und die Infiltration neutrophiler Granulozyten anregen [14].

Auch wenn in eukaryotischen Zellen grundsätzlich reverse Transkriptaseaktivität beispielsweise in Form von LINE‑1 („long interspersed nuclear elements class 1“) vorhanden ist, wurde bei der Anwendung von mRNA-Impfstoffen eine Integration in das Wirtsgenom bisher nicht beobachtet. Aufgrund der geringen Stabilität und der nicht vorhandenen Replikationskompetenz der mRNA ist grundsätzlich davon auszugehen, dass die Bildung des Spike-Proteins nur für eine sehr begrenzte Zeit erfolgt. Es gibt auch Berichte, welche die Nachweisbarkeit von Spike-Protein-codierender mRNA bzw. Spike-Protein in lymphatischem Gewebe über Wochen beschreiben. Allerdings bleibt offen, ob es sich um RNA- bzw. Proteinfragmente oder um die jeweils intakten Moleküle handelt [15].

Auf der Basis der bedingten Zulassung durch die Europäische Arzneimittel-Agentur (EMA) erfolgt bei Erwachsenen die Grundimmunisierung mit BNT162b2 durch 2 Impfungen im Abstand von mindestens 3 Wochen mit je 30 µg RNA, im Fall von mRNA-1273 durch 2 Immunisierungen mit jeweils 100 µg RNA im Abstand von 4 Wochen. Weitere Impfungen („Boosterimpfungen“; 3. oder ggf. 4. Immunisierung) erfolgen frühestens 3–4 Monate nach der abgeschlossenen Grundimmunisierung im Fall von BNT162b2 mit 30 µg RNA und im Fall von mRNA-1273 mit 50 µg RNA.

Die Zulassung für BNT162b2 wurde für Kinder/Jugendliche ab 12 Jahren für die Gabe von je 30 µg erweitert sowie für Kinder zwischen 5 und 11 Jahren auf der Basis einer Gabe von 10 µg RNA. Im Fall von mRNA-1273 können Kinder/Jugendliche ab einem Alter von 12 Jahren mit 100 µg pro Dosis geimpft werden, Kinder von 6–11 Jahren mit 50 µg pro Dosis.

### Adenovirale Impfstoffe

In der EU sind derzeit die auf rekombinanten adenoviralen Plattformen basierenden Impfstoffe AZD1222 (ChAdOx1‑S, Vaxzevria®) von AstraZeneca und Ad26.COV2.S (Jcovden®) von Janssen/Cilag bedingt zugelassen [16, 17].

Bei Adenoviren handelt es sich um unbehüllte Viren mit einem Durchmesser von ca. 80–100 nm, die ein doppelsträngiges, lineares DNA-Genom einer Größe von ca. 26–45 kb (Kilobase) aufweisen. Das Genom weist an beiden Enden hochrepetitive Sequenzen („inverted terminal repeats“, ITRs) auf. Das Kapsid ist aus sogenannten Kapsomeren aufgebaut, wobei Pentone und Hexone zu unterscheiden sind. Ein auffallendes Strukturmerkmal von Adenoviren sind die Fiberproteine, die aus dem Kapsid herausragen und für die Rezeptorbindung eine wesentliche Rolle spielen. Integrine, insbesondere alphavbeta3, alphavbeta5 und alpha5beta1 sowie CAR (Coxsackie- und Adenovirus-Rezeptor) sind relevante Moleküle für die Bindung von Adenoviren.

Die bei adenoviralen Vektoren als Impfstoffplattform dienenden Viren sind bedingt durch das Fehlen des adenoviralen Gens E1 nicht replikationskompetent. Weiterhin fehlt das virale E3-Gen. Durch homologe Rekombination werden nun E1/3-defiziente Genome hergestellt, bei denen das E1-Gen durch die für das Spike-Protein codierende Sequenz ersetzt ist [18, 19]. Zur Amplifizierung der rekombinanten Genome und zur Verpackung werden stabile Zelllinien verwendet (HEK293, „human embryonic kidney cells“, bei AZD1222 oder PER.C6, „human embryonic retinal cells“, bei Ad26.COV2.S), welche die E1-codierende Sequenz als Transgen enthalten.

Bei der Entwicklung adenoviraler Vektoren, die auf RNA-Viren basierende codierende Sequenzen exprimieren, ist zu bedenken, dass der Lebenszyklus nahezu aller RNA-Viren außerhalb des Zellkerns abläuft und daher die Sequenz nicht hinsichtlich des Vorhandenseins von Spleiß-Signalen optimiert sein muss. Wird eine solche Sequenz aber ausgehend von einem DNA-Virus exprimiert, kommt die entstehende RNA im Zellkern in Kontakt mit Spleiß-Faktoren, sodass ggf. mit dem Auftreten von Spleißen („splicing“) zu rechnen ist [20].

Ein grundsätzliches Problem bei der Anwendung adenoviraler Impfstoffe besteht darin, dass in weiten Teilen der Bevölkerung gegen verschiedene humane adenovirale Serotypen neutralisierende Antikörper vorliegen. Um dem zu begegnen, werden entweder Serotypen mit sehr geringer Prävalenz wie Ad26 als adenovirale Plattform eingesetzt oder nicht humane Adenoviren verwendet. Letzteres ist bei ChadOx1 der Fall, hierbei handelt es sich um ein aus Schimpansen isoliertes Adenovirus. Adenovirale Vektoren fanden bereits bei der Entwicklung des Impfstoffs Zabdeno® (Ad.26.ZEBOV, basierend auf Ad26) gegen das Ebolavirus Verwendung [19, 21].

Adenovirale Impfstoffe sind nicht adjuvantiert. Allerdings stimuliert die Injektion von rekombinanten Adenoviren über TLR9 und cGAS (zyklische GMP-AMP-Synthase) die angeborene Immunantwort. Diese inflammatorischen Signale rekrutieren Immunzellen wie DCs und Makrophagen, die von den rekombinanten Adenoviren infiziert werden können. Dadurch kommt es zur Expression des Transgens (Spike-Protein) und zur Einwanderung dieser Zellen in benachbarte Lymphknoten, wo die Induktion einer B- und T‑Zellantwort erfolgt [22].

Im Unterschied zu den mRNA-Impfstoffen weist AZD1222 am N‑Terminus zusätzlich zur endogenen Signalsequenz noch eine „tissue-plasminogen-activator“-(tPA-)abgeleitete Signalsequenz auf. Weiterhin besitzt das von AZD1222 codierte Spike-Protein im Gegensatz zu den beiden zugelassenen mRNA-Impfstoffen keine stabilisierenden Mutationen im Bereich von aa 986 und 987, sodass keine automatische Stabilisierung in der Präfusionskonformation erfolgt. Allerdings zeigen Untersuchungen von AZD1222-infizierten HeLa-Zellen (Epithelzellen eines Zervixkarzinoms), dass trotz des Fehlens der stabilisierenden Mutation das Spike-Protein in der Präfusionskonformation vorliegt [23].

Ad26.COV2.S weist zusätzlich zu den beiden Mutationen an aa 986 und 987 noch eine Mutation der Furin-Spaltstelle (aa682-685) auf, um die Umwandlung in die Postfusionskonformation zu verhindern.

AZD1222 besitzt derzeit eine bedingte Zulassung für die Anwendung bei Personen ab 18 Jahren. Die Immunisierung erfolgt durch 2 Impfungen im Abstand von 4–12 Wochen mit jeweils 5 × 10e10 adenoviralen Partikeln. Für Personen unter 18 Jahren besteht keine Zulassung.

Ad26.COV2.S ist ebenfalls nur für Personen ab 18 Jahren zugelassen. Die ursprüngliche bedingte Zulassung sieht eine einmalige Impfung mit 5 × 10e10 adenoviralen Partikeln vor. Aufgrund von Effektivitätsdaten legen verschiedene NITAGs („national immunization technical advisory groups“) eine Boosterimpfung nach Immunisierung mit Ad26.COV2.S nahe.

### Untereinheiten-Impfstoff

NVX-CoV2373 (Nuvaxovid®) von Novavax basiert auf einem kompletten Spike-Protein, das rekombinant in Sf-9-Insektenzellen produziert wird. Dafür werden die Insektenzellen mit rekombinanten Baculoviren, welche die genetische Information für das komplette Spike-Protein tragen, infiziert. Das Spike-Protein weist zur Stabilisierung der Präfusionskonformation die beiden Mutationen K986P und V987P sowie die Deletion der Furin-Schnittstelle auf.

Durch Detergens-abhängige Solubilisierung wird das Spike-Protein aus den Membranen herausgelöst. Weitere Reinigungsschritte beinhalten 0,2 µm Filtration, Anionentauscherchromatografie, Nanofiltration und Affinitätschromatografie. Das gereinigte Spike-Protein liegt als Trimer in der Präfusionskonformation vor und weist durch die unterschiedliche, an 22 Stellen mögliche Glykosylierung eine Heterogenität hinsichtlich seines Molekulargewichts auf. Da das gereinigte Protein über die C‑terminale Transmembranregion verfügt, erfolgt die Solubilisierung durch Polysorbat 80. Die Transmembranregion insertiert dabei in Polysorbat-80-Mizellen und bildet ca. 27 nm große Nanopartikel, welche aus bis zu 14 Spike-Trimeren aufgebaut sind [24, 25].

NVX-CoV2373 ist ein adjuvantierter Impfstoff. Als Adjuvans dient Matrix M. Dabei handelt es sich um ein neuartiges Adjuvans auf Saponin-Basis. Aus dem fraktionierten Extrakt der Rinde des Seifenrindenbaums (Quillaja saponaria) werden die Fraktionen A und C verwendet, die die Komponenten Matrix A und C in Form von Nanopartikeln enthalten. Weitere Bestandteile von Matrix M sind phosphatgepufferte Salzlösung (PBS), Cholesterol und Phosphatidylcholin [26, 27]. Saponin ist auch Bestandteil der Adjuvanzien ISCOM und AS01B.

Die fertige Formulierung des Impfstoffs enthält je Dosis 5 µg Spike-Protein. Gemäß der bedingten Zulassung ist eine zweimalige Applikation im Abstand von 3 Wochen für Personen ab 12 Jahren vorgesehen.

### Inaktivierte Ganzviren

Seit Juni 2022 ist VLA2001 von Valneva in der EU bedingt zugelassen. Die Verwendung inaktivierter, kompletter Viren als Impfstoff hat eine lange Tradition. Exemplarisch kann hier die von Jonas Salk in den 50er-Jahren des vorigen Jahrhunderts entwickelte Impfung gegen Kinderlähmung gesehen werden. Die Inaktivierung erfolgte im Fall dieses Polioimpfstoffs durch Formaldehyd (Formalin), heute wird – wie bei VLA2001 – häufig Beta-Propiolacton verwendet, das als elektrophiles Agens an Aminogruppen bindet [28].

Trotz der jahrzehntelangen Erfahrung bei der Herstellung und Anwendung von inaktivierten Ganzvirus-Impfstoffen bestanden bei der Entwicklung von COVID-19-Impfstoffen auf Basis inaktivierter kompletter Viren Sorgen, dass es zu einem VAED („vaccine associated enhanced disease“) kommen könnte, analog zu Beobachtungen bei einer früheren Studie zur Entwicklung eines Impfstoffs gegen respiratorische Synzytial-Viren (RSV). Präklinische Daten aus Experimenten zu SARS-CoV-1- und MERS-Impfstoffen schienen darauf hinzudeuten, dass es durch eine ausgeprägte Typ-2-T-Helferzellen-(Th2-)Polarisierung und verstärkte Zytokinbildung zu einem solchen Verlauf kommen könnte. Weder die durchgeführten Studien zu diesem Impfstoff noch Beobachtungen bei anderen in der EU nicht zugelassenen Ganzvirus-Impfstoffen bestätigten diese Befürchtungen [29, 30].

Die Amplifizierung von SARS-CoV‑2 erfolgt in Vero-Zellen, die Isolierung nach Inaktivierung mit Beta-Propiolacton. Im Unterschied zu den Vektorimpfstoffen, die nur auf dem Spike-Protein basieren, beinhaltet das Inaktivatvirus naturgemäß alle viralen Strukturproteine, sodass grundsätzlich von einer breiteren Immunantwort gegen weitere virale Proteine auszugehen ist.

Die Grundimmunisierung erfolgt durch 2 Impfungen im Abstand von 4 Wochen bei Erwachsenen zwischen 18 und 50 Jahren. Eine Dosis des Impfstoffs enthält 33 Antigeneinheiten sowie 0,5 mg Alum und 1 mg CpG1018. Alum und CpG1018 fungieren als Adjuvanzien [31]. Während Alum bereits seit Jahrzehnten als Adjuvans verwendet wird, ist CpG1018 ein vergleichsweise neuer Adjuvanstyp, der beispielsweise auch in dem Hepatitis-B-Virus-Impfstoff Heplisav B® Verwendung findet. Es handelt sich bei CpG1018 um ein unmethyliertes, 22 Nukleotide umfassendes Oligomer. CpG1018 bewirkt eine deutliche Th1-Polarisierung der Immunantwort [27]. Zwar fördert CpG1018 die Reifung von DCs, hat jedoch keinen wesentlichen Einfluss auf die Freisetzung von Interferon (IFN-)α. An Partikel gebundenes oder partikuläres CpG kann in endosomale Strukturen aufgenommen werden und löst über TLR9-abhängige Signalwege die IFN-α-Produktion aus.

## Wirksamkeit

Die Wirkung der Impfung (Verhinderung der Infektion, transiente Infektion und Verhinderung aller Krankheitsverläufe) basiert je nach Impfstoffkonzept nicht nur auf der Induktion einer neutralisierenden Antikörperantwort, sondern auch auf der Induktion der zellulären Immunität. Letztere spielt vor allem eine Rolle bei der Vermeidung schwerer Verläufe und wird insbesondere durch spezifische CD8+-T-Zellen vermittelt.

Besonders mit dem Auftreten der besorgniserregenden Virusvarianten („variants of concern“, VOCs) zeigte sich, dass die Impfung häufig keine sterile Immunität bedingt, sodass es trotz Impfung zu Infektionen und damit auch Weitergabe der Infektion kommen kann, insbesondere bei inapparenten, d. h. symptomfreien Verläufen. Besonders bei dem Ursprungsisolat und den VOCs Alpha, Beta und Delta zeigte es sich, dass es in solchen Fällen häufig zu einem rascheren Abklingen der Infektion kommt [32, 33].

Mit dem Auftreten weiterer Varianten kommt es zu immer größeren Unterschieden zwischen dem in der Regel als Impfantigen verwendeten Spike-Protein des Ursprungsisolats und dem Spike-Protein der zirkulierenden Variante. So zeigen beispielsweise Seren grundimmunisierter Personen eine sehr geringe Neutralisationswirkung gegen die Omikron-Varianten BA.1, BA.4 und BA.5. Allerdings kommt es durch somatische Hypermutation zu einer Verbreiterung der Immunantwort, was bei erneuter Antigenexposition zu einer besseren Neutralisationswirkung auch gegen diese Varianten führt. Diese Antikörper unterscheiden sich jedoch qualitativ (Titer) und quantitativ (Stabilität des Antigen-Antikörper-Komplexes) von den Antikörpern gegen das Ursprungsisolat [34].

Im Rahmen der Zulassungsstudien wurden im ersten Zeitraum nach der Impfung sehr hohe Wirksamkeiten („vaccine efficacy“, VE) von über 90 % insbesondere für die beiden mRNA-Impfstoffe hinsichtlich der Vermeidung von Infektionen gefunden. Naturgemäß sinken diese Werte mit der Zeit teilweise ab, bedingt durch einen Abfall des Titers neutralisierender Antikörper. Wichtig dabei ist jedoch, inwieweit es zur Ausbildung Spike-spezifischer Memory-B- und T‑Zellen (Gedächtniszellen) kommt und wie die Spezifität dieses Zellpools sich verändert. Unmittelbar nach Zulassung der mRNA-Impfstoffe Ende 2020/Anfang 2021 wurden in Israel systematisch Impfungen sehr breiter Bevölkerungsgruppen durchgeführt, zunächst vor allem bei Menschen höheren Alters. So zeigte sich im Frühsommer 2021 in Israel hauptsächlich bei älteren Personen ein Nachlassen der VE insbesondere hinsichtlich der Vermeidung von Infektionen [35, 36]. Dieses Nachlassen, auch als „waning immunity“ beschrieben, ist bei älteren Personen aufgrund von Immunoseneszenz stärker ausgeprägt und kann durch eine Restimulation im Rahmen einer Boosterimpfung revertiert werden.

Mit dem Auftreten weiterer VOCs kam hinzu, dass durch die zunehmende antigenische Distanz zwischen dem der Immunisierung zugrunde liegenden Antigen (Ursprungsisolat) und den zirkulierenden VOCs eine verminderte VE hinsichtlich der Vermeidung von Infektionen beobachtet werden konnte. Dies konnte beispielsweise gut im Zusammenhang mit dem Auftreten der Delta-Variante in den USA beobachtet werden [37]. Besonders ausgeprägt ist diese „waning immunity“ seit dem Auftreten der Omikron-Varianten: Seren komplett immunisierter Personen weisen hier nur eine sehr geringe Neutralisationswirkung auf. Erst durch eine Boosterimpfung kann eine Erhöhung des Titers neutralisierender Antikörper beobachtet werden [34].

Der Effekt der Boosterimpfung basiert hierbei vor allem auch darauf, dass neben der Titererhöhung durch die Restimulation auch die durch somatische Hypermutation entstandenen Omikron-spezifischen Memory-B-Zellen stimuliert werden können und so eine Verbreiterung der Immunantwort beobachtet werden kann [38, 39]. Dies ist jedoch ein zeitabhängiger Prozess, sodass nicht allein die Zahl der Impfungen, sondern vor allem auch der zeitliche Abstand zwischen Abschluss der Grundimmunisierung und Boosterimpfung von entscheidender Bedeutung für die Verbreiterung der Immunantwort ist.

Die zunehmende antigenische Distanz zwischen Ursprungsisolat und der derzeit zirkulierenden VOC Omikron bedingt eine verminderte VE hinsichtlich der Vermeidung von Infektionen, allerdings ist weiterhin eine hohe VE hinsichtlich der Vermeidung schwerer Verläufe zu beobachten. Aufgrund der zahlreichen Mutationen im Bereich des Spike-Proteins kommt es zur Zerstörung einer Reihe von Bindungsstellen für neutralisierende Antikörper, die dadurch entweder nicht mehr oder deutlich schwächer binden. Dies bedingt wiederum ein häufigeres Auftreten von Durchbruchsinfektionen [40, 41]. Da aber auch im Fall der Omikron-Varianten die meisten der T‑Zellepitope noch konserviert sind, kann durch die zelluläre Immunantwort die Infektion noch kontrolliert werden, sodass es häufig zu einer schnelleren Eliminierung der Infektion und weniger schweren Verläufen kommt.

## Zulassung

Die Entwicklung und Zulassung von Impfstoffen gegen COVID-19 in der EU erfolgte teilweise innerhalb von weniger als einem Jahr.

Welche Faktoren trugen dazu bei? Grundsätzlich gibt es in der EU 3 Verfahrenstypen, die eine frühzeitige Zulassung ermöglichen:

- das beschleunigte Bewertungsverfahren („accelerated assessment“),
- die bedingte Zulassung („conditional marketing authorization“),
- die Zulassung unter außergewöhnlichen Umständen („authorization under exceptional circumstances“).

Die Zulassung aller derzeit in der EU zugelassenen COVID-19-Impfstoffe erfolgte als bedingte Zulassung. Eine bedingte Zulassung ist unter bestimmten Bedingungen möglich. Dies beinhaltet, dass der Vorteil der zeitnahen Verfügbarkeit des Arzneimittels die möglichen Risiken einzelner weniger umfangreicher Datenpakete als im regulären Zulassungsverfahren deutlich überwiegt. Ein weiterer Aspekt ist, dass es sich um die Prävention/Behandlung einer lebensbedrohlichen Erkrankung handelt und der Ausschuss für Humanarzneimittel (CHMP) feststellt, dass eindeutig ein positives Nutzen-Risiko-Verhältnis vorliegt und ein bis dato ungedeckter medizinischer Bedarf gegeben ist. Der Antragsteller ist verpflichtet, umfassende Daten zu einem späteren Zeitpunkt vorzulegen. Erfolgt dies nicht, kann die bedingte Zulassung widerrufen werden. Grundsätzlich sind bedingte Zulassungen für ein Jahr befristet, können aber jeweils jährlich verlängert werden und bei Vorliegen aller Datenpakete in eine reguläre Zulassung übergehen [42, 43].

Um die Zulassung von COVID-19-Impfstoffen bestmöglich zu beschleunigen, wurde das Rolling-Review-Verfahren eingesetzt, welches für solche pandemischen Gesundheitslagen etabliert wurde.

Im Rahmen des Rolling-Review-Verfahrens erfolgt die Bewertung und Klärung von Fragen zu den Datenpaketen nicht auf der Basis eines kompletten Pakets von klinischen und nichtklinischen Daten, sondern von nach und nach eingereichten Datenpaketen durch Rapporteure und Co-Rapporteure (federführende Gutachter aus 2 Mitgliedstaaten) des CHMP [44]. Dies bedeutet, dass die Bewertung von nichtklinischen Daten und Daten zur Herstellung und Qualität bereits erfolgen kann, bevor die klinischen Daten aus den Studien vorliegen. Auch für die Genehmigung klinischer Studien wurde das Rolling-Review-Verfahren angewandt. Es erlaubt eine deutliche Verkürzung des Verfahrens, bedeutet aber nicht, dass Abstriche bei den der Bewertung zugrunde liegenden Qualitätskriterien gemacht werden. Es gilt auch im Fall der bedingten Zulassung, dass diese nur dann erteilt werden kann, wenn der Nachweis der Qualität, Unbedenklichkeit und Wirksamkeit erfolgt ist.

Daneben haben auch weitere Faktoren zu der bisher nie erreichten Schnelligkeit bei der Zulassung der COVID-19-Impfstoffe beigetragen. Ein wesentlicher Aspekt ist dabei der sogenannte Scientific Advice durch die Arzneimittelbehörden. Dadurch wird sichergestellt, dass von der frühen Phase der Entwicklung an regulatorische Vorgaben für die spätere Genehmigung klinischer Studien und Zulassung berücksichtigt werden, sodass sichergestellt sein sollte, dass die erhobenen Datenpakete den jeweiligen regulatorischen Anforderungen entsprechen.

Die Kombination einzelner Phasen von klinischen Studien (Phase 1 mit Phase 2 oder Phase 2 mit Phase 3) war ein weiterer Faktor, der wesentlich zur Beschleunigung beigetragen hat.

Auch wenn BNT162b2 und mRNA-1273 die beiden ersten zugelassenen mRNA-Impfstoffe sind, so bestanden aus früheren Entwicklungen zu anderen Erregern oder auf dem Gebiet der Tumorimpfstoffe bereits eine umfangreiche Expertise und Datensätze zu dieser Plattformtechnologie, wie auch für die adenoviralen Vektorimpfstoffe. Auch waren Ergebnisse aus Studien zur Entwicklung SARS-CoV-1- bzw. MERS-spezifischer Impfstoffe hilfreich, um darauf aufbauen zu können. Dazu kommt die bei allen Beteiligten, aufseiten der Entwickelnden wie aufseiten der Zulassenden, hohe spezifische Expertise, die wesentlich zur Entwicklung und deren Beschleunigung beigetragen haben.

## Fazit

Die Anwendung von Plattformtechnologien, die Einbeziehung der Arzneimittelbehörden bereits ab der frühen Phase der Entwicklung im Rahmen des Scientific Advice sowie die Flexibilisierung des Zulassungsverfahrens bzw. der Genehmigung klinischer Studien durch die Anwendung des Rolling-Review-Verfahrens konnten ganz wesentlich zur Verkürzung des Entwicklungs- und Zulassungsprozesses beitragen. Dies bedeutet aber nicht, dass Abstriche bei den der Bewertung zugrunde liegenden Kriterien hinsichtlich Qualität, Sicherheit und Wirksamkeit gemacht wurden.
